# Evaluation and Validation of a Joint Stress Test to Induce Activity-Related Knee Joint Discomfort — a Prospective Case-Control Study

**DOI:** 10.1186/s40798-021-00317-7

**Published:** 2021-04-06

**Authors:** Christiane Schön, Claudia Reule, Katharina Knaub, Antje Micka, Manfred Wilhelm, Wilfried Alt, Daniel Menzel

**Affiliations:** 1 BioTeSys GmbH, Schelztorstraße 54-56, 73728 Esslingen, Baden-Württemberg Germany; 2 Department of Mathematics, Natural and Economic Sciences, Ulm University of Applied Sciences, Albert-Einstein-Allee 55, 89081 Ulm, Baden-Württemberg Germany; 3 Institute of Sports Science and Kinesiology, University of Stuttgart, Allmandring 28, 70569 Stuttgart, Baden-Württemberg Germany

**Keywords:** Knee joint, Activity-related joint discomfort, Joint stress test, Single-leg-step-down test, Range of motion

## Abstract

**Background:**

The assessment of improvement or maintenance of joint health in healthy subjects is a great challenge. The aim of the study was the evaluation of a joint stress test to assess joint discomfort in subjects with activity-related knee joint discomfort (ArJD).

**Results:**

Forty-five subjects were recruited to perform the single-leg-step-down (SLSD) test (15 subjects per group). Subjects with ArJD of the knee (age 22–62 years) were compared to healthy subjects (age 24–59 years) with no knee joint discomfort during daily life sporting activity and to subjects with mild-to-moderate osteoarthritis of the knee joint (OA, Kellgren score 2–3, age 42–64 years). The subjects performed the SLSD test with two different protocols: (I) standardization for knee joint discomfort; (II) standardization for load on the knee joint. In addition, range of motion (ROM), reach test, acute pain at rest and after a single-leg squat and knee injury, and osteoarthritis outcome score (KOOS) were assessed.

In OA and ArJD subjects, knee joint discomfort could be reproducibly induced in a short time interval of less than 10 min (200 steps). In healthy subjects, no pain was recorded. A clear differentiation between study groups was observed with the SLSD test (maximal step number) as well as KOOS questionnaire, ROM, and reach test. In addition, a moderate to good intra-class correlation was shown for the investigated outcomes.

**Conclusions:**

These results suggest the SLSD test is a reliable tool for the assessment of knee joint health function in ArJD and OA subjects to study the improvements in their activities. Further, this model can be used as a stress model in intervention studies to study the impact of stress on knee joint health function.

## Key Points

- The single-leg-step-down (SLSD) test is an appropriate test to induce joint discomfort in subjects with activity-related joint discomfort (ArJD) of the knee.
- The test shows a good test-retest reliability when applying the test for two standardization approaches: (I) standardization for pain level with focus on endpoints of mobility, (II) standardization for load on the knee joint with focus on endpoints of joint discomfort.
- The SLSD test is able to discriminate between healthy subjects (healthy), subjects with ArJD of the knee, and subjects with osteoarthritis (OA) of the knee joint.
- These results confirm the suitability of the SLSD test in intervention studies to study the impact of stress on knee joint health function

## Background

Joint discomfort and joint overload are a common problem not only in daily life of athletes, but also in daily life of recreationally active people. These symptoms can limit mobility and flexibility also in healthy people without diagnosed joint diseases, like osteoarthritis (OA) or rheumatoid arthritis. The functional joint discomfort occurs because of short-term degradation of cartilage and increased stress on the joints [1–3]. Stress-related joint pain can be caused by anatomical weaknesses (e.g., unequal leg length, bandy legs, small patella), mechanical overload, or unstable joints as a result of earlier ligament injuries. In such cases, the homeostasis of cartilage building and cartilage degradation processes loses balance under load and discomfort occurs for a certain period. These conditions and symptoms occur long before manifestation of a disease like osteoarthritis [4, 5].

Measuring an improvement or maintenance of joint health in healthy subjects is a great challenge. A collective of people with activity-related joint discomfort (ArJD) but without any diagnosed knee joint diseases is a potential cohort for observation and evaluation of joint health. Some research groups already worked with people with ArJD and their research showed promising results [6, 7]. However, in these cases, pain was only evaluated under daily life activity and regular sporting activity. Daily life activity is characterized by variable intensities and therefore by variable load on the joints resulting in different stress levels for joint related structures. For the clinical evaluation of supplement effects in ArJD, it is highly relevant to (a) exclude as many external influencing factors as possible and (b) lower the variability to increase the chance of significance.

Therefore, stress models (joint or muscle related) have been used in the past to induce standardized load on physical structures to be able to observe changes on perceived stress as accurately as possible [8–10]. For the observation of knee joint function, different joint bearing movements are qualified to be used during a stress model. A treadmill test or downhill walking are joint bearing movements already used in different approaches; however, these kinds of evaluations have a high cardiopulmonary component possibly limiting the performance of the tests [8, 11]. Furthermore, a variety of costly equipment is necessary, which can limit the test usability.

Aim of the study was the evaluation and specification of a joint stress test to evaluate joint discomfort in healthy subjects with ArJD of the knee compared to healthy subjects without ArJD and subjects with diagnosed mild-to-moderate OA as control groups. An easy test set up with a targeted load on the knee joint without high impact on cardiovascular fitness was a precondition for the test protocol. Focus of the clinical set up was set on symptomatic endpoints as well as on joint function. The supportive assessment of systemic biomarkers was not focus of the study. To qualify a test protocol for future studies, the test set up with suitable movements needed to (a) induce high mechanical load on the knee joint, (b) be suitable to differentiate between study groups, (c) show high degree of reproducibility, (d) be easy to carry out, (e) show no or low training effects, and (f) offer a time-efficient approach to induce joint discomfort without harm on joint structures.

A step-down movement was finally selected for the study set up, as this movement has the highest biomechanical load on the knee joint. During walking upstairs, the knee joint is loaded with up to 3.0× of the body weight and during walking downstairs with up to 3.5× of the body weight [12–14].

In the present study, the joint stress test was performed with respect to two standardization approaches:

1. Standardization for joint discomfort, allowing to measure the impact on joint mobility.
2. Standardization for load, allowing to measure the impact on joint discomfort and investigate parameters of recovery.

## Methods

### Study Design

The study was a prospective, 3-arm, non-interventional study at the study site of BioTeSys GmbH (Esslingen, Germany). The study was conducted in accordance with the guidelines for Good Clinical Practice (GCP) set forth by the International Conference on Harmonization of Technical Requirements for Pharmaceuticals for Human Use (ICH), and the Declaration of Helsinki regarding the treatment of human subjects in a study.

During day 1 (test 1), after 14 days (test 2) and after 28 days (test 3), subjects had to do a joint stress test. A detailed test procedure was defined for each subject individually. The range of motion (ROM) and most distance walk during a reach test were recorded to characterize the study group. Acute pain at rest and after a single-leg squat on an 11-point numeric rating scale (NRS-11) at different time points pre- and post-joint-stress-test (only test 2 and test 3) were determined. The assessment of the study parameters was done on the leg with usually more intense pain after physical activity (target knee). In healthy subjects, the target knee was the non-dominant leg, which was assessed with a shove-test. In OA patients, the target knee was the knee with higher pain. During the 2 weeks prior to test 2 and test 3, subjects recorded their sporting activities in a diary and documented if knee pain occurred during activity. Average knee pain during regular sporting activity was recorded using NRS-11.

To reduce the effect of confounding factors, study subjects were asked to maintain their usual diet during the study time. Further, subjects were not allowed to use any pain medication (e.g., aspirin, paracetamol) or supplements influencing joint pain. Forty-eight hours before to the test days, the subjects were not allowed to perform any sporting activities or other exhaustive physical activity, like heavy gardening or hiking.

### Subjects

From January to July 2018, subjects were screened for their eligibility at BioTeSys GmbH (Esslingen am Neckar, Germany). Informed consent from each subject participating in the study was obtained after written and oral explanation of the aims, methods, benefits, and potential hazards of the study.

Healthy non-smoking men and women at the age of 18–65 years with a BMI between 19 and 29.9 kg/m^2^ were eligible to participate. Healthy and ArJD subjects had to perform joint bearing sports for at least 2 times per week (e.g., soccer, basketball, handball, volleyball, tennis, running). Subjects with activity-related pain and healthy subjects were matched according to age and BMI. Subjects in the ArJD group additionally had to suffer from reversible knee joint discomfort during or immediately after physical activity over a period of at least 3 months. OA subjects had to have diagnosed with mild or moderate osteoarthritis in the knee without planned surgical treatment (known X-ray finding Kellgren score of 2 to 3). The diagnosis was confirmed by a study orthopaedist prior to the subject’s inclusion in the study.

Exclusion criteria included joint replacement of the knee, planned surgical intervention during the study conduct, intra-articular therapy within the last 3 months prior to the study start and relevant history or presence of any medical disorder potentially interfering with this study, like active cancer, heavy cardiovascular diseases (e.g., stroke, heart attack) as well as pregnancy and lactation. Additionally, subjects in the OA group were excluded if they had diagnosed rheumatoid arthritis or other inflammatory, infectious, or metabolic joint disorders except OA. Subjects from the healthy and ArJD group were neither supposed to have acute knee pain at rest or during daily life activities nor having acute or a history of musculoskeletal injury or previous severe joint injury at the knee joints (e.g., ligament rupture or surgical intervention of meniscus), if not completely recovered without remaining functional impairment and if not more than 2 years ago. Subjects with hip, spine, and foot pathology were also excluded from study participation.

Only those subjects with no identified exclusion criteria were invited to take part at screening (test 1).

### Objectives

#### Joint Stress Test/Single-Leg-Step-Down Test

The single-leg-step-down (SLSD) test is an unilaterally performed test used in literature as a functional performance test in patients with patellofemoral pain syndrome [12]. As the movement stresses the whole knee joint due to the high mechanical load, a modification of this test for the evaluation of the actual load capacity of the knee joint was used.

**Fig. 1:**
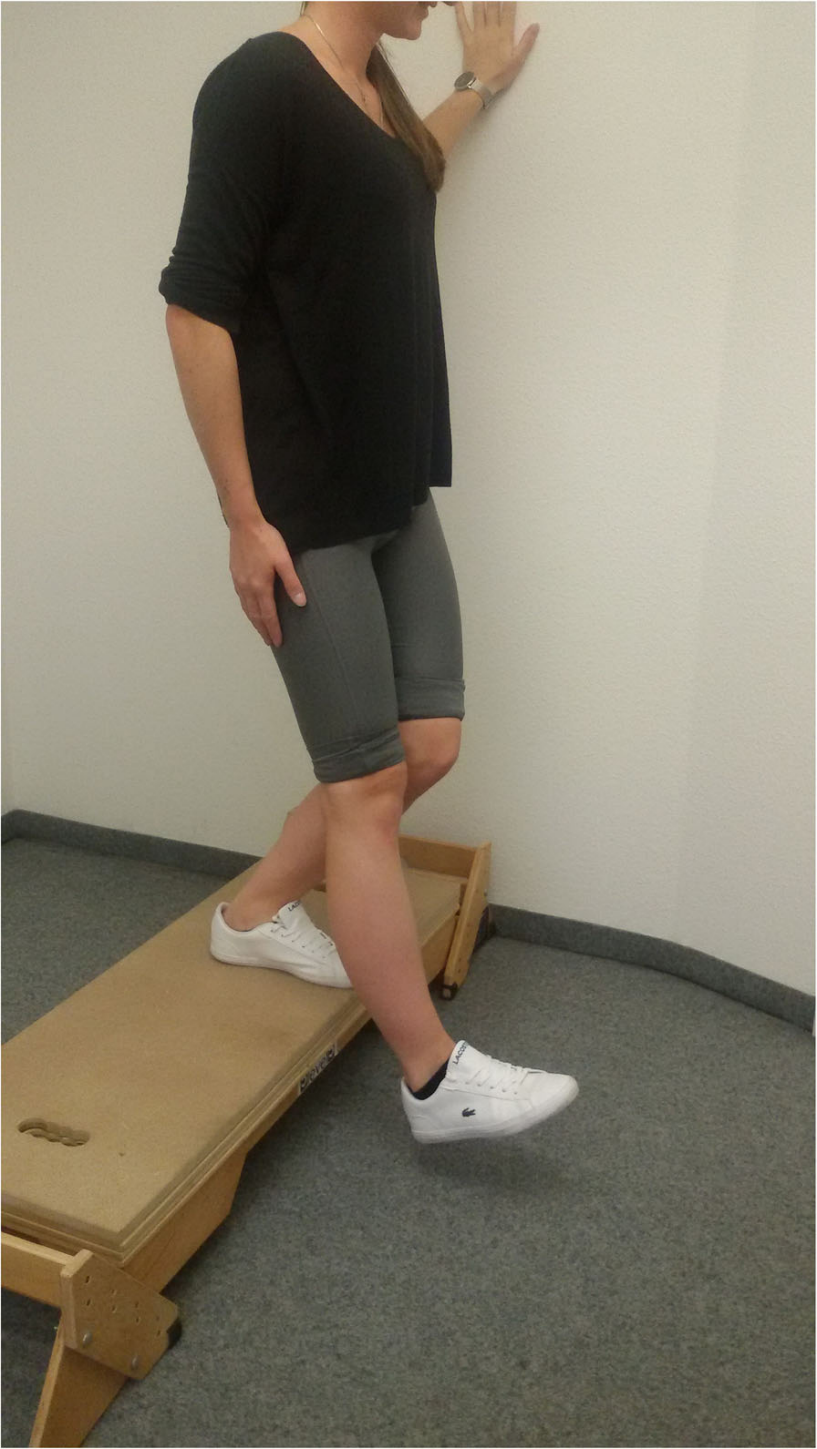
Single-leg-step-down test

Subjects had to step forward and down from a platform with adjustable height. The down limb only brushed the floor with the heel and then returned back up to the platform to full knee extension. Figure 1 provides the illustration of the test performance. The height of the platform was adjusted on the length of the subject’s shank. The height of the step for subjects with a shank length of, e.g., 46 cm was 20 cm. Based on this, the height of the step was adjusted by dividing the individual shank length by 2.3. The frequency of repetitions was given by a metronome. Subjects were instructed to show the pain level of 2 while performing the test and to stop the test as soon as they experience a pain level of 5 on NRS-11, where 0 meant “no pain” and a pain of 10 meant “worst pain possible”. The number of total repetitions was recorded. If no joint pain occurred after at least 10 min, the stress test was not continued. The steps were performed with a time interval of 3 s allowing a maximum of 200 steps.

**Fig. 2:**
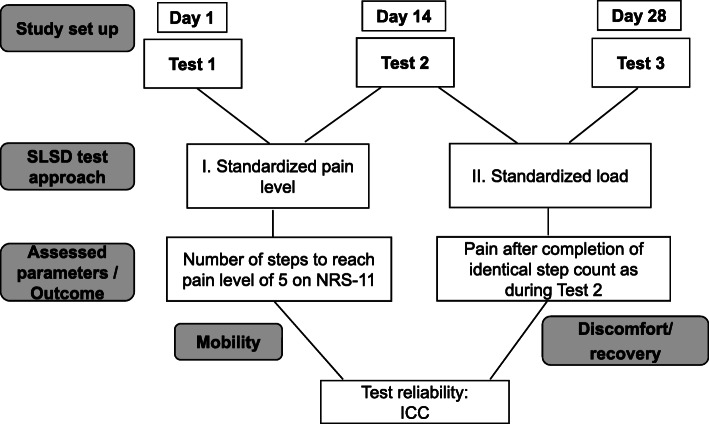
Overview over the study set up for the groups OA and ArJD. Abbreviations: ArJD: activity-related joint discomfort, OA: osteoarthritis, SLSD: single-leg-step-down, ICC: intra-class correlation, NRS-11: 11-point numeric rating scale

The step test was conducted three times with two different approaches:

1. During test 1 and test 2, subjects performed the test until they experienced a pain of 5 on NRS-11. Additional aim of this set up was to derive cut-off values for the number of steps to discriminate between OA and ArJD.
2. During test 3, subjects performed the same number of steps as during test 2. The maximum discomfort was assessed to investigate if the movement induces a comparable discomfort level by same load. After test 2 and test 3, acute joint discomfort was additionally monitored to test the impact on recovery. Figure 2 provides the schematic illustration of the test set up. It is important to note, that this set up is only applicable for the study groups OA and ArJD, as the subjects of the healthy group were not expected to experience any pain during the performance of the SLSD test.

#### Range of Motion

A joint’s ROM, used to assess the joint flexibility, is usually measured by the number of degrees from the starting position of a segment to its position at the end of its full range of movement. In the current study, range of motion measurement was assessed by using a digital goniometer (Halo Medical Devices). The positioning of the goniometer was determined by the two most prominent bone protrusions of the lower leg. They include the lateral condyle of the femur to determine the knee joint centre and the lateral malleolus for the ankle joint.

For the measurement subjects lied in prone position with shanks hanging free over the edge of the examination couch in maximal extension. Active ROM was assessed by measurement of the maximum possible flexion in the knee joint. To measure the passive ROM, the flexion of the knee was performed by examiner pressing against the upper shank to the maximum possible flexion.

ROM measurement was performed prior the stress test performance.

#### Reach Test

**Fig. 3:**
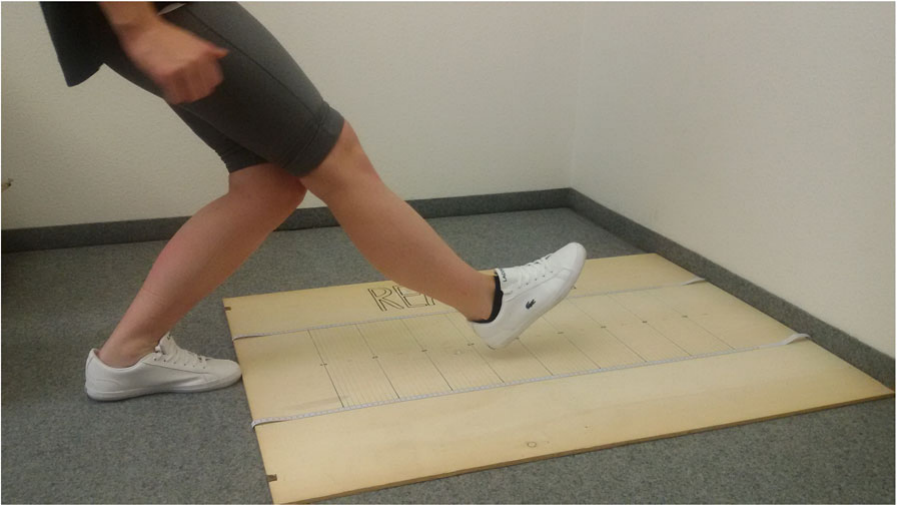
Performance of the reach test

The reach test was adapted based on the balance and reach test [12]. The subject was standing in an upright position with the toe of the target leg behind the start line. The subject flexed the target knee and reached straight forward as much as possible with the non-target leg in full extension, so that the heel of the latter touched the floor. The distance from the toe of the target leg until the heel of the other leg was measured. As maximal distance depends on shank length, data were corrected for shank length to allow a better direct comparison between groups. Figure 3 provides the illustration of the test performance. The reach test was performed prior to the stress test.

#### Retrospective Joint Discomfort (KOOS)

The KOOS (knee injury and osteoarthritis outcome score) questionnaire is used for knee injury that can result in post-traumatic osteoarthritis; i.e., ACL (anterior cruciate ligament) injury, meniscus injury, and chondral injury [15, 16]. KOOS consists of 5 subscales: pain, other symptoms, activity in daily living (ADL), function in sport and recreation (Sport/Rec), and knee-related quality of life (QOL).

For the retrospective measurement of joint discomfort during daily life and physical activity, the KOOS was completed prior to the stress test.

#### Acute Joint Discomfort

Acute joint discomfort was assessed at rest at timepoints prior, immediately after (0 h), 0.5 h, 2 h, 4 h, 6 h, 12 h, and 24 h after the stress test (only test 2 and test 3). Further, joint pain was additionally assessed while performing a single-leg squat (subjects were allowed to support themselves on a table with one hand). For this purpose, a 10-cm visual analog scale (VAS) with 0 no pain and 10 maximal imaginable pain was used.

#### Joint Discomfort During Regular Sports

Between the test days, subjects had to document the intensity of pain felt during or immediately after their normal sports activities using NRS-11, as well as the duration of pain after sports in a diary. Average duration of knee pain was also assessed via diary and mean values were calculated from the reporting. In ArJD group, the time until full recovery from pain was additionally reported.

### Methods for Safety

During the study, the subjects documented any adverse events (AEs) and concomitant medication in diaries. These entries were monitored and judged by the delegated study staff.

Further, blood routine parameters and vital signs were assessed prior to test 1. Blood pressure and heart rate were determined after 5 min of rest in a sitting position.

If parameters of blood routine and vital signs were evaluated as clinically not relevant, subjects were allowed to participate.

### Data Collection

Source documents were filled in by authorized staff to collect information about the compliance to study standards, adverse events, study progress and efficacy parameters, and data was afterwards transcribed into the electronic case report form (eCRF).

### Statistical Analysis

The current study was planned as pilot study for evaluation of a suitable joint stress test for further research studies and to demonstrate test-retest reliability with *n* = 15 in each target group. For test-retest reliability, within each study arm, the intra-class correlation coefficient (ICC) was calculated. For the assessment of intra-class correlation, the two-way mixed model was used with type absolute agreement. To describe precision, the 95% confidence interval (CI) was calculated. For interpretation of ICC, the guideline by Koo and Li 2016 was followed with ICC < 0.5: poor, between 0.50 and 0.75: moderate, between 0.75 and 0.90: good and above 0.90: excellent ICC [17].

Furthermore, within each study arm results from joint stress test for approach (I) standardization for joint discomfort (test 1 vs. test 2) and (II) standardization for load (test 2 vs. test 3) were analyzed using paired *t* test or Wilcoxon signed rank test, as appropriate. Acute joint discomfort was investigated within each study arm with baseline corrected results from joint stress test using a linear mixed model with repeated measures and the appropriate baseline level as covariate.

Changes within each study test and study arm for parameters with repeated measures were analyzed with repeated measurement ANOVA or Friedman test.

Comparison of study arms was performed for assessments at test 2 using one-way ANOVA or Kruskal-Wallis test. In case parameters could not be determined in the healthy control group, unpaired *t* test or Wilcoxon rank sum test was applied.

All statistical tests were performed two-sided. If not indicated otherwise, a significance level of 0.05 was used. Statistical analysis was performed using the software Prism Version 5.4, SPSS 24.0 and SAS 9.3

## Results

### Subjects Characteristics

**Fig. 4:**
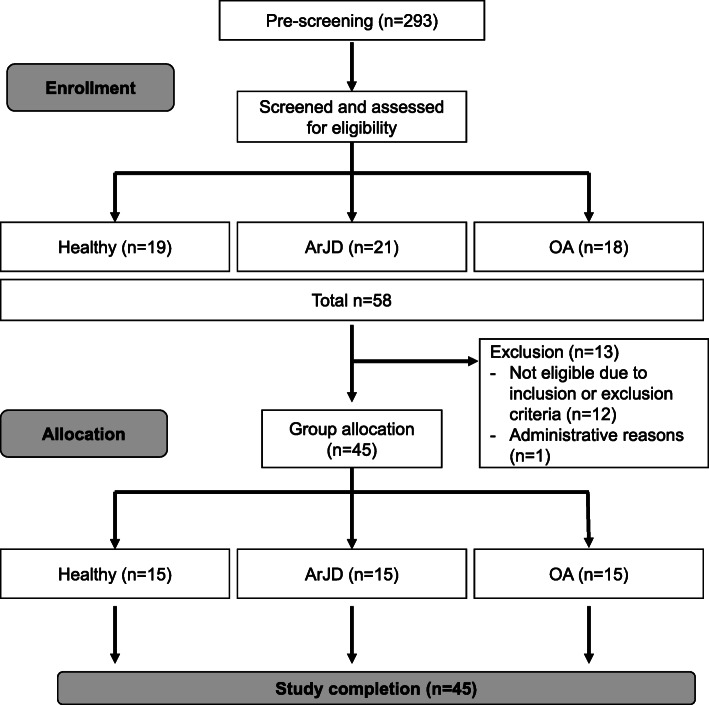
Flow chart of subject recruitment. Abbreviations: SLSD: single-leg-step-down, ArJD: activity-related joint discomfort, OA: osteoarthritis

Out of 58 screened subjects, 45 subjects were enrolled in the study. There were no drop outs during the study and all subjects completed the study in its entirety. The overview of the subject disposition as well as primary reasons for non-inclusion of screening failures (4× subjects applied for healthy group, 6× subjects applied for ArJD, and 3× subjects for OA) are summarized in Fig. 4.

Overall, 25 women and 20 men participated in the study. The sex breakdown was evenly represented in each group. Subjects in the OA group were significantly older in comparison to the ArJD (*p* < 0.001) and the healthy group (*p* < 0.001). Baseline characteristics of the study subjects at the screening timepoint are presented in Table 1.

**Table 1:** Demographics and baseline characteristics

Variable		Healthy (***n*** = 15)		ArJD (***n*** = 15)		OA (***n*** = 15)	
		Mean	95% CI	Mean	95% CI	Mean	95% CI
Age [years]		**37.7**	30.6 to 44.9	**37.5**	30.5 to 44.6	**54.7**	50.9 to 58.6
Weight [kg]		**70.3**	63.9 to 76.7	**73.1**	64.9 to 81.2	**74.0**	67.9 to 80.1
Height [m]		**1.74**	1.69 to 1.78	**1.74**	1.69 to 1.79	**1.73**	1.69 to 1.77
BMI [kg/m^2^]		**24.6**	23.1 to 26.2	**24.0**	22.6 to 25.4	**23.2**	21.8 to 24.7
Blood pressure sys [mmHg]		**126.2**	116.4 to 136.0	**124.3**	116.3 to 132.4	**129.1**	121.2 to 136.9
Blood pressure dia [mmHg]		**75.2**	69.1 to 81.3	**73.2**	68.0 to 74.8	**82.7**	77.1 to 88.3
hsCRP [mg/L]		**0.1**	0.05 to 0.13	**0.1**	0.06 to 0.14	**0.3**	0.09 to 0.76
Total cholesterol [mg/dL]		**183.3**	165.8 to 200.9	**181.8**	168.0 to 195.6	**200.0**	182.8 to 217.2
Gender [*n*]		***n***	**%**	***n***	**%**	***n***	**%**
Male		**7**	46.7	**7**	46.7	**6**	40.0
Female		**8**	53.3	**8**	53.3	**9**	60.0

Values are means (95 % CI) except for qualitative variables (*n*). Abbreviations: *ArJD* activity-related joint discomfort, *OA* osteoarthritis, *BMI* body mass index, *sys* systolic, *dia* diastolic, *hsCRP* high sensitivity C-reactive protein

In the OA group, only subjects with mild-to-moderate osteoarthrosis as confirmed by X-ray were eligible to participate. Eleven subjects were diagnosed with Kellgren score 2 and 4 subjects with Kellgren score 3. Majority of subjects of this group suffered from knee pain since more than 2 years.

### Sporting Activities

Overall, all subjects in the study groups healthy and ArJD reported to regularly perform sporting activities (at least twice per week). In the OA group, only two subjects stated not to do regular sports.

Subjects in the ArJD group reported to have knee joint discomfort for more than 3 months (60% of subjects at least 1 year) but only during the regular sporting activities, especially during knee joint bearing sports like badminton, cycling, fitness, handball, running, and soccer. The majority of subjects however reported to have recovered from pain at rest within 1–2 h after the ending of the sporting activities.

### KOOS Questionnaire

KOOS questionnaire was filled in by all subjects. As expected, subjects in the healthy study arm showed nearly no impairment in the different subscales. For the ArJD, subject’s impairment was identified and the OA group showed the strongest symptoms (see Table 2). A clear differentiation was seen between the OA group and the ArJD group as well as the healthy group regarding all KOOS subscales. There were also some significant differences between the healthy and ArJD group, particularly regarding the subscales pain (*p* < 0.01), function in sport and recreation (*p* < 0.01) and knee related quality of life (*p* < 0.01).

**Table 2:** Baseline data for ROM, reach test and KOOS subscales

Parameters	Healthy (***n*** = 15)		ArJD (***n*** = 15)		OA (***n*** = 15)	
	Mean	95% CI	Mean	95% CI	Mean	95% CI
Reach test	**1.022**	0.949 to 1.095	**0.984**	0.911 to 1.056	**0.821**	0.761 to 0.881
ROM active [°]	**132.9**	126.9 to 138.9	**129.2**	123.6 to 134.8	**117.0**	107.8 to 126.2
ROM passive [°]	**151.9**	146.4 to 157.3	**147.7**	140.7 to 154.8	**127.3**	116.1 to 138.5
**KOOS subscales**						
Pain	**100.0**	100.0 to 100.0	**86.0**	80.4 to 91.7	**65.0**	55.9 to 74.1
Symptoms	**97.3**	95.3 to 7.2	**88.5**	81.4 to 95.7	**68.5**	58.8 to 78.1
ADL	**99.9**	99.7 to 100.1	**96.1**	92.6 to 99.7	**77.1**	67.4 to 86.7
Sport/Rec	**99.7**	99.0 to100.4	**78.0**	71.7 to 84.4	**45.3**	33.2 to 57.5
QOL	**100.0**	100.0 to 100.0	**69.2**	61.1 to 77.3	**42.6**	34.5 to 50.7

Values are means (95% CI). *Abbreviations*: *ArJD* activity-related joint discomfort, *OA* osteoarthritis, *ROM* range of motion, *ADL* activity in daily living, *Sport/Rec* sport and recreation, *QOL* quality of life.

### ROM

Baseline values for ROM were comparable between the healthy and ArJD group, indicating that subjects from ArJD did not suffer from function impairment. Significant differences in ROM active and passive were seen between healthy and OA (ROM active *p* < 0.01; ROM passive *p* < 0.001) as well as ArJD and OA (ROM active *p* < 0.05; ROM passive *p* < 0.001) (see Table 2)*.*

ROM measurement was a good and reliable parameter to characterize the different study groups.

### Reach Test

**Fig. 5:**
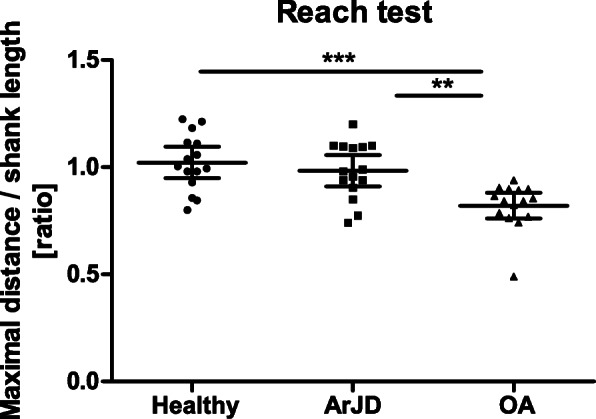
Assessment of maximal distance during reach test corrected for shank length comparing healthy, ArJD, and OA group. Scatter plot with mean and 95% CI; **p < 0.01, ***p < 0.001. Abbreviations: ArJD: activity-related joint discomfort, OA: osteoarthritis

The comparison of the values corrected for the shank length at baseline revealed no differences between the healthy and ArJD group. However, the OA group significantly differed in comparison to the healthy and ArJD group (see Fig. 5).

### Joint Stress Test/Single-Leg-Step-Down Test

The SLSD test was appropriate to induce a pain level of 2 as well as of 5 in the study subjects of both groups, OA and ArJD. The number of steps needed for increase of pain by one score on NRS-11 did not seem to be a linear function and greatly depended on the individual person.

#### Approach I: Standardization for Pain Level

Number of repetitions to reach pain of 5 in different study groups is presented in Table 3.

**Table 3:** Distribution of results of SLSD: steps to reach the pain levels 2, 2 to 5, and 5

Parameters of SLSD	Test 1	Test 2	ICC
**SLSD test [steps] pain 2**			
ArJD (*n* = 15)	21.1 (15.4 to 26.8)	20.5 (14.0 to 27.0)	0.555 (− 0.407 to 0.854)
OA (*n* = 14)	9.9 (6.6 to 13.3)	9.5 (6.7 to 13.3)	0.7 (0.024 to 0.905)
**SLSD test [steps] pain between 2 and 5**			
ArJD (*n* = 15)	34.5 (24.9 to 44.1)	37.7 (28.1 to 47.4)	0.789 (0.382 to 0.929)
OA (*n* = 15)	14.3 (9.1 to 19.4)	13.2 (9.5 to 19.9)	0.737 (0.160 to 0.916)
**SLSD test [steps] pain 5**			
ArJD (*n* = 15)	55.5 (45.0 to 66.)	58.2 (43.9 to 72.5)	0.815 (0.449 to 0.938)
OA (*n* = 15)	24.2 (17.1 to 31.3)	22.1 (16.2 to 28.0)	0.779 (0.352 to 0.926)

Values are means (95 % CI). *Abbreviations*: *ArJD* activity-related joint discomfort, *OA* osteoarthritis, *ICC* intra-class correlation, *SLSD* single-leg-step-down

All healthy subjects performed 200 repetitions without experiencing any knee pain at both tests. In the ArJD group, the mean number of steps necessary between pain 2 and pain 5 was 34.5 (95%CI 24.9–44.1) during test 1 and 37.7 (95%CI 28.1–47.4) during test 2. In the OA group, the mean number of steps between pain 2 and pain 5 was 14.3 (95% CI 9.1–19.4) during test 1 and 13.2 (95% CI 9.5–19.9) during test 2. In one subject of the OA group, the assessment of step count until the pain 2 was not possible as the subject already started the test with pain level of 2.

Subjects of the ArJD group performed significantly more steps in comparison to the OA group. This was confirmed for the pain level of 2 on NRS-11 (*p* < 0.01) as well as for the pain level of 5 (*p* < 0.001).

Overall, ICC for all assessed endpoints was moderate to good. To reach pain level of 5 as well as the number of repetitions needed to get from pain level 2 to 5 was the more reliable parameter with consistent good ICC in both study groups ArJD and OA (see Table 3) in comparison to onset of pain (pain level of 2).

**Fig. 6:**
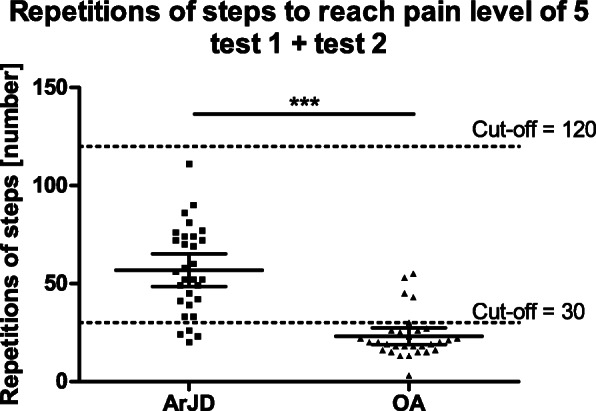
Distribution of repetitions of steps [number] to reach pain of level 5 on NRS-11 in ArJD and OA group at test 1 + test 2; scatter plot with mean and 95% CI; ***p < 0.001. Abbreviations: ArJD: activity-related joint discomfort, OA: osteoarthritis

To define the cut-off levels for the performance of the SLSD test, the data from both tests were averaged. Based on the distribution of the data in ArJD subjects (median: 54 repetitions of steps (25th–75th percentile 41–74)) and OA subjects (median 20 repetitions of steps (25th–75th percentile 16–26)), a lower cut-off value of 30 steps was defined to reliably discriminate between ArJD and OA (see Fig. 6).

#### Approach II: Standardization for Load on the Target Knee

**Fig. 7:**
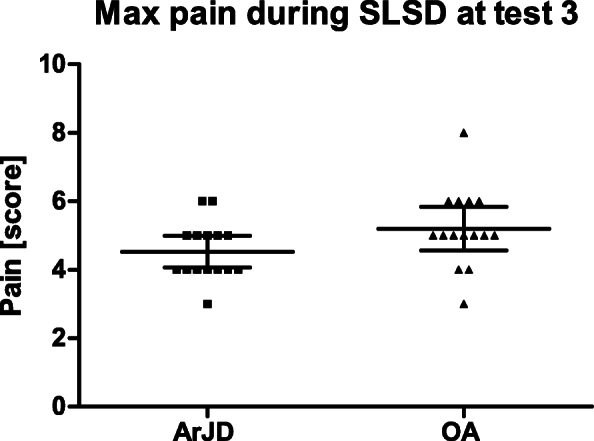
Distribution of maximum pain on NRS-11 [score] while performing the joint stress test in ArJD group; scatter plot with mean and 95% CI. Abbreviations: ArJD: activity-related joint discomfort, OA: osteoarthritis, SLSD: single-leg-step-down

In the second approach, the load was individually standardized based on test 2. When doing the same number of repetitions at test 3, subjects ended on average at a pain level of 4.5 (95% CI 4.1–5.0) in the ArJD group and 5.2 (95% CI 4.6–5.8) in the OA group. In the ArJD group, the pain level at test 3 was slightly lower (*p* = 0.0593). In the OA group, the level was slightly higher (*p* = 0.6082) compared to test 2 (see Fig. 7).

### Acute Knee Joint Discomfort and Recovery

Acute knee joint discomfort was assessed at rest prior to the SLSD test as well as to the timepoints immediately after (0 h), 0.5 h, 2 h, 4 h, 6 h, 12 h, and 24 h after the stress test.

Healthy subjects did not feel any knee pain at rest or during the single-leg squat prior and after the SLSD test. Therefore, no data are presented for the healthy group.

**Fig. 8:**
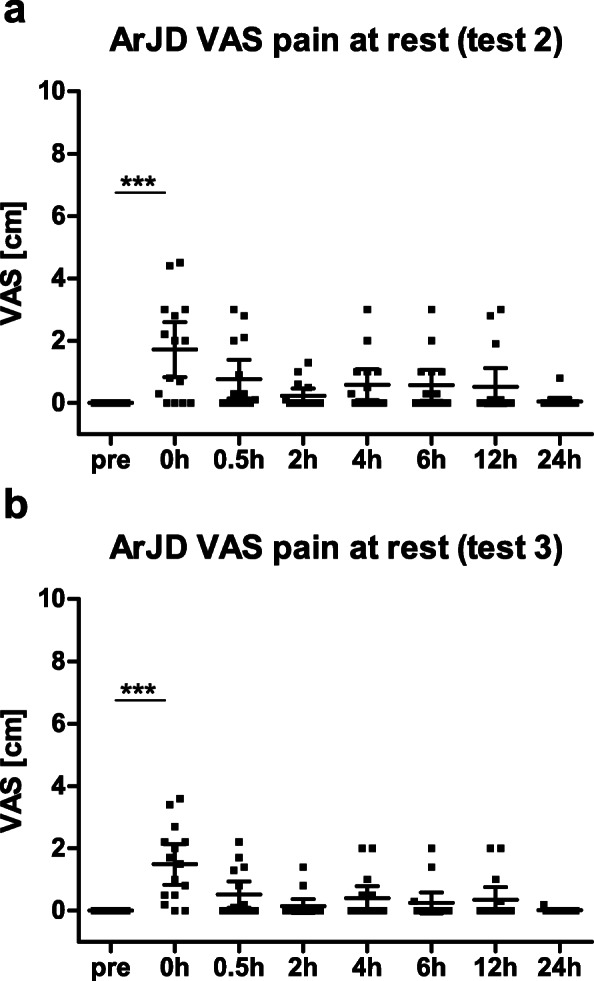
Distribution of pain at rest [cm] assessed via VAS scale in ArJD group pre and post stress test ***a*** at test 2, ***b*** at test 3; scatter plot with mean and 95% CI; ***p < 0.001. Abbreviations: ArJD: activity-related joint discomfort, OA: osteoarthritis, VAS: visual analog scale

Subjects of ArJD group also did not feel any pain at rest. However, when performing a single-leg squat, a low level of pain was caused in 13 of 15 subjects at test 2 during the pre-assessment (mean pain 1.55 (95% CI 0.85–2.25)). The acute joint discomfort was also monitored after the SLSD test. As pain level of 5 was induced by the stress test, subjects also indicated some pain at rest, which was most pronounced directly after the test. Within 2 h after the tests, the majority of subjects recovered from pain at rest (see Fig. 8a, b). Similarly, the pain level when performing a single-leg squat first significantly increased after the stress test and then decreased over time.

Interestingly, the pain level at rest increased again between the time points 4 h and 24 h. This can be explained by the fact, that subject left the study site after the 2 h measurement, so that the load on the knee joint increased due to daily life activity, whereas within the first 2 h after the stress test subjects spend at rest at the study site. In the OA group, 6 of 15 subjects reported to suffer from pain at rest at test 2 with a maximum pain level of 4.6. All subjects felt a pain when doing the single-leg squat (mean pain 3.19 (95% CI 1.89–4.49)). The level of pain during single-leg squat was significantly higher in the OA group in comparison to the ArJD groups (*p* = 0.0242).

**Fig. 9:**
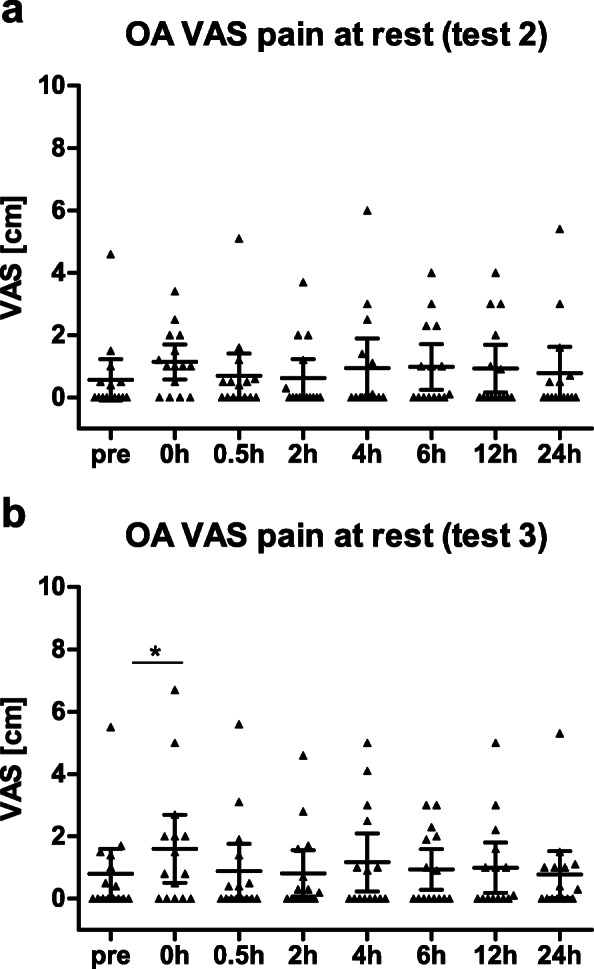
Distribution of pain at rest [cm] assessed via VAS scale in OA group pre and post stress test ***a*** at test 2, ***b*** at test 3; scatter plot with mean and 95% CI; *p < 0.05. Abbreviations: ArJD: activity-related joint discomfort, OA: osteoarthritis, VAS: visual analog scale

In the OA group, also a significant increase over time was seen for pain at rest after the stress test at test 2 and test 3 (see Fig. 9a, b).

The reliability for maximum knee joint discomfort after the stress test was good to excellent for VAS at rest (ArJD: test 2: 1.79 (95% CI 0.94–2.65), test 3: 1.49 (95% CI 0.83–2.14); OA test 2: 1.33 (0.59–2.07), test 3: 1.64 (95% CI 0.50–2.78)), and VAS after the single-leg squat (ArJD: test 2: 2.65 (95% CI 1.95–3.36), test 3: 2.11 (95% CI 1.43–2.80); OA: test 2: 3.69 (2.50–4.87), test 3: 3.57 (95% CI 2.23–4.92)) (ICC see Table 4).

**Table 4:** ICC for maximum knee joint discomfort; test 2 vs. test 3

**Maximum knee joint discomfort at rest**		
**0–2 h**	**ICC**	**95% CI**
ArJD	0.780	0.364 to 0.925
OA	0.732	0.203 to 0.910
**Maximum knee joint discomfort during squat**		
**0–2 h**	**ICC**	**95% CI**
ArJD	0.776	0.342 to 0.924
OA	0.911	0.735 to 0.970

Values are means (95% CI). *Abbreviations*: *ArJD* activity-related joint discomfort, *OA* osteoarthritis, *VAS* visual analog scale, *ICC* intra-class correlation

## Discussion

Activity-related joint discomfort is a common problem in daily life of physically active people, as this may limit their mobility and flexibility as well as physical performance. The discomfort develops as a consequence of overloading or incorrect loading of the joint during physical activity. In this context, the knee joint is the most concerned joint as many sports like soccer, tennis, and jogging pose a high load on the knees. Many athletes report from activity induced joint pain [2]. In the long-term, such imbalances can increase the risk to develop osteoarthritis [4].

Signs of overloading can be influenced by bioactive ingredients. However, to measure the impact of such ingredients standardized tests are necessary. Such stress tests are already implemented for cardiovascular events, e.g., exercise ECG. Further, models of delayed onset of muscle soreness (DOMS) are recognized approaches to study exercise recovery. In the field of joint health, different biomarkers of cartilage were investigated and the expression of several biomarkers was found to be sensitive to mechanical loading [18–21]. However, there is no single marker to assess joint health or functional capacity [22, 23].

Aim of the present study was to set up a stress test with focus on joint health to assess joint discomfort and joint function parameters. In literature, different approaches are described in healthy subjects with joint discomfort like pain during intensive soccer play, change of pain intensity during activity or an aerobic step test with consecutive training at alternating days but with focus to endpoints of cartilage turn over [7, 6, 24]. To our knowledge, for none of these approaches, the reliability of tests was evaluated for the target population of subjects with ArJD.

The SLSD movement is a movement that is very easy to perform by everyone and does not require any special training. It is a movement that poses a high targeted load on the knees without cardiopulmonary exhaustion. Furthermore, the involvement of other muscle groups and their loading to fatigue as well as induction of sore muscles is less pronounced. Even though some other muscle groups are involved in the performance of the step test, i.e., predominantly the hip musculature, the load on the knee joints is paramount. The performance of almost every other conceivable movement (e.g., treadmill, downhill walking over longer time period which is also simulated in the lab by using a treadmill) will require more than one physical performance factor (knee joint, lower body musculature, cardiopulmonary system) possibly limiting the use of the test for different target groups. Additionally, biomarkers might be influenced by such other factors, which complicates interpretation of joint specific parameters. To overcome these limitations, the step-down test was developed. This test seems to be a suitable approach to study joint health specifically by induction of knee joint discomfort under standardized conditions.

The SLSD test could be easily performed by all subjects independent of training status and was therefore suitable for the different subject groups of ArJD and OA. Out of all screened subjects, only 4 subjects could not be included due to endurance or circulatory problems (1× healthy, 2× OA) as well as back pain (1× ArJD) when performing the test. This underlines the suitability of the test for a broad range of subjects.

Another advantage of the SLSD test is that due to high load on the knee, joint discomfort could be reproducibly induced in ArJD and OA subjects within a short period of time, less than 10 min. A maximum level of joint discomfort of 5 corresponding to moderate joint discomfort was selected based on ethical considerations as well as on cut-off levels defined for other sport models [11].

The stress test was performed following a standard protocol with standardized intervals for the steps. The height of steps was adjusted to shank length to better inter-individually standardize the load on joints. The key to the reliability of the test is that standard protocols were followed to keep the impact by observer as low as possible.

### Group characteristics

Prior to the induction of pain by the stress test, the function impairment was additionally measured with different approaches. The range of motion measurement was performed under active and passive conditions. ROM measurement is a widely used method for the assessment of flexibility of different joint types. It is also used for the evaluation of changes of knee joint flexibility in the context of physical or surgical therapy. The findings of the current study support the reliability of the ROM measurement, as both parameters, passive and active ROM, were reliable measures to characterize the study groups. In OA subjects, ROM active and passive was significantly impaired in comparison to the healthy and ArJD groups. However, to measure changes of interventions by nutraceuticals, the parameter might lack sensitivity because of measurement variability.

The same applies to the reach test. This test is an appropriate function test to characterize the different study groups. Differences between the healthy as well as ArJD group in comparison to the OA group were significant. The test is an accepted test also for subjects with patellofemoral pain syndrome. However, to investigate intervention effects in ArJD subjects the test might be not sensitive enough [12].

Additionally, the KOOS questionnaire was used to assess knee joint discomfort at baseline measurement. There was a clear differentiation between the ArJD and OA group. The KOOS questionnaire confirmed in all subscales the clear differentiation between subjects with ArJD and healthy subjects. Further, the KOOS questionnaire indicated that even if the knee pain only occurred during heavy load or sporting activities, this already had some impact on knee related quality of life and especially the subscale sport and recreation. This indicates that the KOOS questionnaire is sensitive enough to cover the impairment in this borderline collective and clearly differentiates between impaired and healthy populations. In a study with amateur football players of mean age of 22 years comparable levels to the ArJD group of our study were reported for the subscale symptoms and ADL [25]. KOOS scores reported for the OA group clearly confirm the impairment; however, the study group of OA subjects in the present study showed high activities of daily living, less pain and thus the quality of life impairment was less pronounced in comparison to a study of Braham et al. [26]. This might also be explained by the fact that the majority of participants in our study only suffered from mild osteoarthritis with Kellgren score 2 (73.3%).

### Reliability of Step Test —  Approach I: Standardization for Pain Level

Within a given time interval, subjects had to perform as many repetitions as possible until a subjective pain/joint discomfort of 5 on NRS-11. This approach focuses on the increase or decrease of mobility. Translated to daily life: if an intervention could increase the number of repetitions until pain 5, this means that more stairs can be climbed or soccer can be played longer before any joint discomfort occurs, which has a great impact on quality of life.

In the ArJD group, the number of repetitions was significantly higher in comparison to OA subjects. Thus, a clear differentiation of ArJD and OA subjects was possible. Therefore, the joint stress test is also suggested as a tool to select subjects for participation in clinical trials. A cut-off value of 30 steps, which is based on the distribution of the data in subjects with activity-related joint discomfort and osteoarthritic subjects, seems to be appropriate to quite reliably discriminate between these two populations. Applying the cut-off value of ≥ 30 steps, sensitivity was calculated with 87% to truly identify subjects with ArJD, with a specificity of 83%.

The data also indicate that the number of repetitions clearly depends on the individual endurance and the kind of sport-related stress the subject is used to, and also explains the overlap between target groups. Interestingly, no impact of age or BMI was seen in our study data, neither in subjects with ArJD nor in the OA group (data not shown). Based on this background as upper cut-off level, 120 to 150 steps are suggested with respect to minimize heterogeneity of subjects but also to allow subjects to participate with great endurance. Such a cut-off level would also allow the determination of intervention success if the maximum step number of 200 from the healthy subjects is applied.

For the healthy subjects, the test was stopped after 200 repetitions. The subjects did not complain from knee pain, but due to the single side movement, some subjects reported back pain and muscle soreness. Taking this under consideration, the study experience confirms the suitability of the upper limit of 200 steps for a healthy and athletic population to keep other confounding factors, e.g., muscle soreness from other muscle groups as low as possible.

Overall, ICC of the SLSD test for number of repetitions in ArJD and OA group was moderate to good. It is commonly known that sportive challenge tests are prone to training effects or adaptation reactions especially with eccentric muscular loads [27]. In contrast, data observed were very comparable between test 1 and test 2 even with a short time period of 2 weeks between the tests with no significant differences neither in subjects with ArJD nor in the OA group and neither for number of repetitions until pain level 2 nor until pain level 5. This means that no training effects were identified, which is a great advantage of the stress test and supports its robustness. Only minor changes between the two tests were observed which could be attributed to normal variability of subjective ratings of discomfort (pain thresholds/ autonomic threshold) and normal physiological variability of reactions responsible to induce the joint discomfort during the step test and normal day-by-day variability of physical fitness.

### Reliability of Step Test — Approach II: Standardization for Load on Target Knee

The second approach was the evaluation of the response of step test on joint function and pain while keeping the load on joints identical between the two repeated measurements (test 2 and test 3).

Keeping the load identical indicated that during both occasions the movement induced a comparable pain/discomfort level. The reported pain range at test 3 was slightly higher in OA group with a range of 3–8 in comparison to ArJD (range 3–6) which may be attributed to the fluctuating symptoms known for OA. Despite the subjective assessment of pain, the reproducibility of the stress test was very good. The induction of pain was confirmed in all participating subjects.

Next to the maximum pain at the end of the stress test, the pain at rest was assessed at specified time points. Healthy subjects and subjects of the ArJD group did not have any pain at rest when they started the stress test. After the stress test, the majority of subjects of the ArJD group indicated some pain at rest, which was most pronounced directly at the end of the step test. Majority of subjects recovered from the pain within 2 h after the test. The reliability for maximum knee joint discomfort after the stress test was good to excellent for pain at rest in both study groups.

Further, the pain level after a single-leg squat was evaluated prior and after the SLSD test. Despite a good reliability between the tests, performing a squat was a challenge for some subjects due to balance issues. Therefore, subjects were allowed to stabilize but only with little force. These test conditions were individually standardized to avoid external impact factors. Anyhow, it turned out that there was some factor of variability with regard to how deep the squat was performed which might have had an impact on the pain level. Therefore, in future assessments, we would highly recommend to better standardize the movement, e.g., with a goniometer.

## Conclusion

The current study could confirm that the SLSD test is a reliable tool for the assessment of knee joint health in subjects with ArJD.

Joint discomfort was induced with an easy to handle movement targeted to the knee joint allowing for high standardization. The test was able to discriminate between different subject groups and showed a good test-retest reliability applying the test with focus to endpoints of mobility as well as endpoints of joint discomfort. Requirements for a stress model were confirmed to allow the use in future intervention studies to measure impact on joint health. Thus, the SLSD test seems to be an appropriate test to measure knee joint function parameters in a healthy population with ArJD by inducing inter-current discomfort. The sensitivity to capture joint health benefits within intervention studies needs to be investigated in future studies and compared to response to other assessments, like quality of life changes or reporting from situations during which activity-related joint discomfort normally occurs.

## Data Availability

The data that support the finding of this study are not publicly available due to privacy or ethical restrictions but are available from the corresponding author on reasonable request.

## Abbreviations

ADL: Activity in daily living
AE: Adverse event
ANOVA: Analysis of variance
ArJD: Activity-related joint discomfort
BMI: Body mass index
BP: Blood pressure
DOMS: Delayed onset of muscle soreness
ECG: Electrocardiogram
eCRF: Electronic case report form
CI: Confidence interval
GCP: Good clinical practice
hsCRP: High sensitivity C-reactive protein
ICC: Intra-class correlation
ICH: International Conference on Harmonization
IRB: Institutional review board
KOOS: Knee injury and osteoarthritis outcome score
NRS-11: 11-point numeric rating scale
OA: Osteoarthritis
ROM: Range of motion
SLSD: Single-leg-step-down
Sport/Rec: Function in sport and recreation
QOL: Quality of life
VAS: Visual analog scale
